# Dr. Beddoes's Case of Hydrophobia

**Published:** 1808-09

**Authors:** Thomas Beddoes

**Affiliations:** Clifton


### Medical and Physical Journal.

VOL. XX.] SEPTEMBER 1808. [NO. 115.

*Printed for R. PHILLIPS, by W. Thorne, Red Lion Court, Fleet Street, London*

### *To the Editors of the Medical and Physical Journal.*

GENTLEMEN,

I Send you two cases, as the first of a series of papers, calculated to throw light on opinions, which have been advanced in modern times, on the cause and seat of several important disorders. Of some of these papers I find myself in possession from the favour of correspondents, and of others from the course of my own practice. The two cases now sent are of the former description. They are distinguished by a variety of curious particulars, and form suitable companions, on account of the similarity of the local treatment. These taken along with the facts, which I have stated in *Researches concerning Fever*, and indeed with the whole mass of accurate evidence, shew pretty clearly how far certain inflamed or sub-inflamed blotches in the stomach, are essential to hydrophobia in the human species. Since the alarm in 1806 and 1807, which if too violent or general, was certainly no false alarm, I have not only chanced to communicate with various practitioners, to whom cases of this terrible disorder had occurred, but endeavoured to prevail upon some others to communicate their observations to the public. Had I not been occupied in drawing up a little piece of *Advice to the Husbandman in Harvest*, and other matters, I might have renewed my solicitations. And I wish that I could draw forth any accurate journals, and dissections, not already in train for publication.

I am, &c.

THOMAS BEDDOES.

*Clifton, Aug.* 19, 1808.

---

John Dyke of Kemberton Quarters, near Shifnal, aged 9 years, was bitten by a dog supposed to be mad, on the 3d of October 1806. The following day I first saw him; the cheek and lip were lacerated in four different places, nearly nearly through their substance. Muriate of antimony was applied to the parts, and this application alternately with argentum nitratum was continued at intervals for nine days, as often as the eschars were thrown off, when he neglected coming to be dressed, and the parts healed.

On Monday the 27th of October I saw him again, and learnt that he had been shivering, and poorly, for part of two days. He complained of a pain in his throat, and at the pit of his stomach. His tongue was white, the tonsils streaked with slight inflammatory lines, but not enlarged. His eyes appeared heavy, and the cornea suffused with blood in a slight degree; pulse 108, skin hot, countenance dejected. Respiration at times natural, but frequently interrupted with deep inspirations amounting to sobbing; but without any disposition to shed tears. There was resemblance to that quick mode of respiration which is produced on going into a cold bath in a gradual manner, when the water first reaches the scrobiculus cordis. He had not any sickness: but the accumulation of saliva in the mouth produced an effort to expel it, similar to the action of vomiting; and this occurred as often as a fresh accumulation took place. I offered him a little cold water to drink, when immediately the same convulsive respiration took place. When he took the cup he held it at arm's length in both hands for a few seconds, as though afraid to carry it to his mouth, which he did at length with a sudden impulse; but the instant the fluid entered his mouth, his eyes started forwards from their sockets, his shoulders and chest were elevated as if he were determined to force it down his throat, but all in vain. He spat it out with great vehemence, and appeared much agitated for some time afterwards. Upon offering him a little *warm gin and water*, the same symptoms occurred, but with less violence; and he suffered a little to pass the fauces. Upon being asked to describe the part that hurt him when swallowing, he pointed to his throat below the thyroid cartilage, and said that it also hurt him at the pit of the stomach. He had no idea whatever of the nature of his complaint; which indeed was carefully concealed from him. When a mirror was presented to him, he complained in a few seconds of its hurting his eyes. The same convulsive sobbing took place, as on the attempt to swallow water, and he turned his head aside with great expression of fear. I gave him money to induce him to look at it a second time, and endeavoured to engage his attention by desiring him to point out to me by the mirror, which of the sores had given him the greatest uneasiness at the time of dressing them; but before fore he had looked in it a minute, the same effect was produced as before; and upon asking him why he did not continue to look at it, he said, "it made his eyes feel like his cheek did when the burning spirits were put on it."—The cicatrices looked of a redder hue than would be thought natural, and felt a little elevated; but he did not complain of soreness upon pressure. He could swallow bread and butter; and also took a grain of opium in a pill without much difficulty; the pill was ordered to be repeated every six hours, and he was put in a warm bath up to his chin, which produced great tranquillity of countenance, and he said he could breathe much easier. Whilst in the water, he drank a tea-cup full of warm milk and water, but not without great eagerness and starting of the eyes, but with much less difficulty than before. His bowels had been perfectly open for several days.

Tuesday 28th. Symptoms much the same, but he had passed a restless night, owing to the disturbance he experienced from the saliva passing to the fauces, none of which he was able to swallow, and it was become very viscid.—The warm bath was repeated. Clysters of milk, sugar, and broth, were administered frequently; and the pills continued as before. He could still swallow a little *warm* liquid, but not without great difficulty. Upon going into the air, he complained of the cold affecting his throat and stomach, and it brought on the same sobbing as before; which was also the case whenever he was asked to drink any thing, though the liquid was not presented to him.

Wednesday 29th. Had passed a restless night; and the attendants said he had had several fits, but was perfectly sensible when out of them; other symptoms as before. Continue the glyster, baths, and opium.

Thursday 30th. Several fits during the night; was delirious, with few lucid intervals; talked incoherently of "*the waggon passing over his throat and belly*;" shewed increased affection for those who waited upon him, but still exhibited marks of distrust and fear at every thing they were doing around him. He had not past any fæces for three days past. His belly was become very hard, and much distended. Gave him 5 grains of calomel in a pill, which he swallowed very readily, and afterwards half a spoonful of Castor oil in as much broth. But this he did not get down without great difficulty. Ordered the oil and the clysters to be repeated; and to discontinue the opium till his bowels were relieved.

Friday 31st. Pulse, which had continued with little variation, was now become more feeble. Tongue very white; almost constant delirium; but still sensible enough to distinguish persons, and repeat the Lord's Prayer correctly, which he did voluntarily. Countenance disturbed at every attempt to swallow; but still able to get down a little broth and Castor oil; and in proportion as debility increased, the spasmodic action was diminished. Urine involuntarily; no stool; continued the clyster and Castor oil.

Saturday, Nov. 1st. Plentiful evacuations from the bowels; abdominal tumour subsided; other symptoms as before; but debility more apparent.

Sunday, Nov. 2d. Messrs. Bayley, Stanin, jun. and Bennett, saw him in the morning; he was still sensible enough to put out his tongue when asked to do so; but appeared sinking very fast. A mirror was again shewn him, but he appeared insensible to its effects. When I saw him in the evening, he was quite insensible; his pulse scarcely distinguishable; clammy perspirations; and about five o'clock, expired without a struggle. He never shewed the least vicious propensity to his attendants during his illness; nor uttered any of those peculiar sounds which have been described to occur in this malady.

Nov. 4th. Dissection. Upon opening the larynx and trachea, the internal membrane appeared considerably inflamed, and covered with a frothy mucus, slightly tinged with blood, extending from the glottis downwards, to the bronchiæ. The upper and anterior surface of the lungs appeared flaccid, and of a natural colour; but upon turning up the left lobe, it appeared considerably higher coloured than the anterior surface; and the pleura also appeared in a state of inflammation. The under surface of the right lobe and the pleura appeared in a similar state, but in a less degree. The heart and large blood-vessels appeared in their natural state. On opening the pharynx, the internal membrane appeared considerably inflamed; but the inflammation did not extend at all down the œsophagus, which, together with the stomach, appeared in their natural state, as did also all the abdominal viscera, except that the texture of the stomach was less firm than usual. The tongue was white and furred. The tonsils, uvula, and velum pendulum palati, did not appear at all affected. The head was not opened.

### *Mr. Richard Smith's Case of Hydrophobia.*

Daniel Perry, aged 7, a native of Westbury under the Plain, Wiltshire, was admitted into the Bristol Infirmary by Mr Richard Smith, the 17th of Sept. 1807. Two days before, he had been bitten by a dog supposed to to be mad, and the day after the accident had been taken to Kingroad and dipped in the salt water, as is usually done by the common people. The teeth of the animal had wounded the eye-lid slightly, and made a laceration upon the forehead, about two inches long.

The parts were severely cauterized with the calx cum kali puro, and a bread and milk poultice was applied. Due attention was paid to the bowels, but no other medicine was administered.

The sloughs having been thrown off at the expiration of five days, the granulations were immediately *destroyed again* by the paste caustic as before, and this was repeated *six times*; in fact, as often as the parts destroyed by the application were thrown off, and the os frontis almost denuded.

The lad continued cheerful, played about the ward, and took his meals with the patients without the least inconvenience during the whole of this time.

On the 21st of October, which was 34 days from the date of his admission, and 36 from the time of the accident, the lad's stomach appeared disordered, and he was ordered viii grains of ipecacuhana: he vomited, and was relieved.

On the 22d, he complained of pain in his forehead and of thirst; was languid, and made some efforts to vomit; his pulse was 126, and the pupil very much dilated; he could swallow fluids with a little difficulty; he took the mixt. salina edulcorata, and seemed better towards evening. Letters from the lad's father within a few days, having induced us to think that the dog had not been mad, we hoped that these symptoms were merely the commencement of a fever.

But on the morning of the 23d, all the symptoms were aggravated; his pulse rose to 140, his tongue was white, the fauces very red, and the difficulty of swallowing so great that he refused liquids altogether.

His breathing was laborious, and made a rattling kind of noise as if there was a fluid in the trachea; fifteen drops of opium were administered.

At mid-day the lad became suspicious, screamed out if any one attempted to come near him, and seemed in an agony if the wound was touched, or even blown upon.

He had not, however, lost his reason; and feeling uncomfortable at the frequent convulsive movements of his body, wished to be tied or held fast.

Any afforts to drink brought on violent affection of the muscles of deglutition, but there was a remarkable

 difference difference between attempts to drink hot or cold liquids; the convulsions produced by the latter were terrible.

At times the boy seemed not to suffer at all; he talked incessantly about his playmates, was very happy, eating cake and apple with little difficulty.

At half past 3 o'clock, P. M. his pule was 108. At 4 o'clock the temporal artery was opened, and the jugular vein, but only a few ounces of blood could be obtained; pulse rose under the bleeding to 130; the blood exhibited no marks of inflammation. A blister was applied round the throat; two drachms of mercurial ointment, camphorated, were rubbed into his thigh, and the wound sprinkled with powdered opium, the patient having previously undergone the operation of the shower bath.

He complained of being very cold and thirsty, called for water, and drank a small quantity pretty well, but soon after screamed and struggled violently.

One drachm of the powdered leaves of digitalis purpurea was macerated for an hour in six ounces of boiling water, half an ounce of which liquid was given with 15 drops of tinct. of opium.

At 5 and 6 o'clock there had been considerable reaction upon the system, but he was so feeble that the pulse was not discernible in the extremities.

At 7, he sat up, drank his warm tea with the nurse without difficulty, eat some cake, and seemed greatly mended.

At 8, the other temporal artery was wounded, but the blood would not flow; he was again placed under the shower bath, but he soon sunk to the floor perfectly exhausted, and was carried into bed.

At 10, he seemed again rather mended, but in half an hour had two violent spasms, and some singultus.

At half past 11, the shower bath was again used, but the patient soon fainting, he was wiped dry and carried to bed. Pulse not perceptible.

At 12, he was seized with an involuntary motion of the head, which continued 20 minutes.

At 20 minutes before one, he was offered some tea, and appeared willing to drink it, but had not the power to swallow it.

At half past one, (now the 24th) 15 drops of tinct. opii. were administered, in some warm tea, with considerable difficulty, several slight spasms intervening, and an hour elapsed before he could be persuaded to swallow the whole; the latter part gave him the least uneasiness.

At 2, he made a strange noise twice, resembling a good deal the mewing of a cat; in 20 minutes he swallowed a table spoonful of warm tea, without producing spasm.

At

At half past 3, he began to be extremely restless.

At 4, his extremities were clammy and cold; and at 26 minutes after 4, there was a convulsion of the whole body.

At 40 minutes after 4, he became quite delirious and very restless, extremities being quite cold.

At a quarter past 5, there were several slight spasms, accompanied with groans; he now ceased to talk.

At 25 minutes past 5, subsultus tendinum came on, and in a few minutes the boy expired, without any violent symptom.

On the same day at 1 o'clock, P. M. the body was examined by Mr. Smith, in the presence of the physicians, surgeons, and students belonging to the Institution.

The pharynx, œsophagus, and trachea, exhibited no preternatural appearances, except that there was rather an unusual quantity of mucus about them.

The stomach and intestinal canal were empty, and both perfectly free from all morbid appearance, with the exception of the ileum, upon the villous coat of which the blood vessels seemed rather turgid.

The mucous membrane of the bladder seemed slightly inflamed near the cervix.

In all other respects the thoracic, abdominal, and the pelvic vicera were in every part free from any disorganization.

The dura mater exhibited unequivocal marks of inflammation, chiefly in the direction of the longitudinal sinus.

The pia mater, the substance of the cerebrum and cerebellum, the cavities, mednlla oblongata and naves, exhibited nothing worthy of remark; and, in fact, with the exception of the dura mater, there was nothing in the whole examination, which, under other circumstances, would have arrested the attention.

The facts are here simply stated, and the medical reader will form his own opinion as to the nature of this horrible disease. *The antiphlogistic plan, in this case, was pushed very far, and failed of success.*

---

